# "Costal Resistance" in Abdominal and Thoracic Diseases

**Published:** 1904-05-21

**Authors:** 


### "COSTAL RESISTANCE" IN ABDOMINAL AND THORACIC DISEASES.

There are certain points to be noticed about the behaviour of the costal arch in affections of the abdomen and thorax that are of value, according to Dr. E. Eliot,[1] as diagnostic symptoms. The costal arch, composed of the consecutive portions of the seventh, eighth, ninth, and tenth costal cartilages, in health participates in the respiratory movements of the chest and abdomen, being slightly elevated during inspiration, and lowered during expiration. The normal range of movement, however, is frequently disturbed, and this is true, not only of the active respiratory motion, but more especially of that passive movement toward the abdominal cavity which the costal arch may be made to describe by the pressure of the examiner's hand.

Interference with the active respiratory excursions is observed by inspection, and is manifested chiefly by a difference in extent of expiration on the two sides, although if the pathological condition be painful the deficient movement will be present on both sides.

To estimate the "costal resistance" the fingers of the two hands should be placed at corresponding segments of the costal arch, and gentle pressure made simultaneously, during expiration, in a direction toward the vertebral column, and any difference in degree of mobility of either arch carefully noted. In the presence of an underlying neoplasm the sensation imparted to the palpating hand is of a hard, unyielding nature, and may be described as "board-like" as distinct from elastic. If on the other hand there be an acute or sub-acute inflammatory condition present, the costal resistance is very highly elastic, and the excursion of its movement considerably limited. Dr. Eliot claims for this mode of examination (1) that asymmetry of costal resistance may be detected when there is no marked difference in the respiratory excursions of the costal arches; (2) in inflammatory lesions of the lower part of the abdomen, increase of costal resistance will indicate extension of the disease process to the upper part of the abdomen; (3) in inflammatory lesions of the upper part of the abdomen a difference in degree of costal resistance on the two sides will be a valuable guide to localisation of the abdominal trouble.

[1] Medical News, April 23.

